# Elevated red cell distribution width predicts residual dizziness in patients with benign paroxysmal positional vertigo

**DOI:** 10.3389/fneur.2022.857133

**Published:** 2022-09-01

**Authors:** Ke-Hang Xie, Li-Chun Chen, Ling-Ling Liu, Chu-Yin Su, Hua Li, Run-Ni Liu, Qing-Qing Chen, Jia-Sheng He, Yong-Kun Ruan, Wang-Kai He

**Affiliations:** ^1^Department of Neurology, Zhuhai Hospital of Integrated Traditional Chinese and Western Medicine, Zhuhai, China; ^2^Department of Encephalopathy, Shantou Hospital of Traditional Chinese Medicine, Shantou, China; ^3^Department of Nephrology, The Fifth Affiliated Hospital of Sun Yat-sen University, Zhuhai, China; ^4^Department of Neurology, Zhuhai Hospital of Integrated Traditional Chinese and Western Medicine, Zhuhai, China

**Keywords:** residual dizziness, benign paroxysmal positional vertigo, red cell distribution width, oxidative stress, inflammation

## Abstract

**Objective:**

The present study aimed to determine whether residual dizziness (RD) after successful repositioning treatment in benign paroxysmal positional vertigo (BPPV) patients could be predicted by red blood cell distribution width (RDW).

**Materials and methods:**

In this study, a total of 303 BBPV patients hospitalized at the neurology department were investigated. The enrolled patients were divided into two groups after successful repositioning treatment: non-RD group included patients who were completely cured, and RD group included patients with RD. We collected data on all subjects, including general information, blood routine examination, blood biochemical examination, and magnetic resonance imaging tests.

**Results:**

The mean RDW values of patients in the RD group were significantly higher than that in the non-RD group (13.63 ± 1.8 vs. 12.5 ± 0.8; *p* < 0.001). In subsequent multivariate analysis, elevated RDW levels were a statistically significant risk factor associated with the occurrence of RD [odds ratio = 2.62, 95% confidence interval (CI) 1.88–3.64, *p* < 0.001]. The area under the ROC curve was 0.723 in terms of its predictive ability to distinguish patients with RD. A cut-off point of 12.95% of RDW predicted RD with a sensitivity of 75.6% and a specificity of 69.5%. Moreover, the AUC for the ability of the RDW to predict recurrence were 0.692 (95% CI = 0.561–0.831; *p* < 0.014).

**Conclusions:**

Elevated RDW level was related to increased risk of RD among BPPV patients, requiring further efforts to clarify the actual underlying pathophysiology.

## Introduction

Up to 31%~61% of benign paroxysmal positional vertigo (BPPV) patients suffered from residual dizziness (RD) in the first few days or weeks after successful canalith repositioning procedures (CRPs) (1, 2). Characterized by dizziness, balance disorders, or unsteadiness, RD could seriously affect older adults' gait and balance and increase their risk of falling and consequent injuries (3).

The exact pathophysiology of RD remains controversial (4–7). Oxidative stress (OS) is known to play an important role in several inner ear diseases (8–10). Previous studies have shown that BPPV patients suffered from higher levels of OS than healthy groups (11). In addition, after complete recovery of BPPV symptoms, the index of the OS status decreased significantly (12). Therefore, it can be inferred that the reduction of OS in patients with BPPV after treatment is parallel to symptomatic relief. Whether there is a difference in OS status between RD and non-RD after treatment in BPPV patients has rarely been studied.

The red cell distribution width (RDW) is a biochemical parameter representing the variability in size of circulating red blood cells, which is considered as an informative clinical marker outside of anemia assessment in routine practice (13). Elevated RDW is associated with the occurrence, development, and prognosis of central nervous system diseases (14–16). OS and subsequent subclinical inflammation may be important pathophysiological mechanisms of this clinical phenomenon as increased RDW comprehensively represents a higher level of OS damage (17, 18). Recent experimental evidence has shown that the vestibular system is very sensitive to oxidative damage (12, 19). Therefore, it is compelling to speculate that the alteration of RDW level may be a predictive indicator of RD in BPPV patients. As far as we know, no study has focused on the relationship between RDW and RD.

In this study, we aimed to investigate whether RDW value was helpful in the prediction of RD after successful CRPs in patients with BPPV.

## Materials and methods

### Participants

Thorough history, neurological, and magnetic resonance imaging (MRI) examinations were performed on all BPPV patients at the neurology department of Zhuhai Hospital of Integrated Traditional Chinese and Western Medicine between December 2014 and December 2020. In this study, only patients with posterior semicircular canal lithiasis (PSC-BPPV) were included and treated with the Epley maneuver. For patients with severe cervical spondylosis or obesity, which could not be coordinated with Epley's maneuver, Semont maneuver was used for reduction.

All patients underwent a bedside neurological assessment, including examination of a mixed torsional and upward beating nystagmus during the Dix-Hallpike position, vestibulo-ocular reflex, and optokinetic and balance tests (20). During the evaluation period, the patients also underwent diagnostic location testing for BPPV assessment. On the second week following successful CRPs, the enrolled patients were subsequently divided into two groups (based on the results of the second Dix-Hallpike test): non-RD group included 509 patients without any dizziness, and RD group included 186 patients with persistent and non-positional atypical dizziness (absence of true positional vertigo and nystagmus). The characteristics of nystagmus in the enrolled patients were observed and recorded by Danish Vestibular nystagmus view analyzer VN415/VO425. In addition, the observation of nystagmus was performed with the patient wearing a blindfold and in a dark room to eliminate the effects of fixation suppression.

Participants also had to meet the following inclusion criteria: (1) no central diseases (including central vertigo), cranial traumas, or stroke; (2) no anemia, immunologic diseases erythrocytosis, cancer, infection, severe cardiovascular disease, and hepatic or renal impairment.

**Figure 1:**
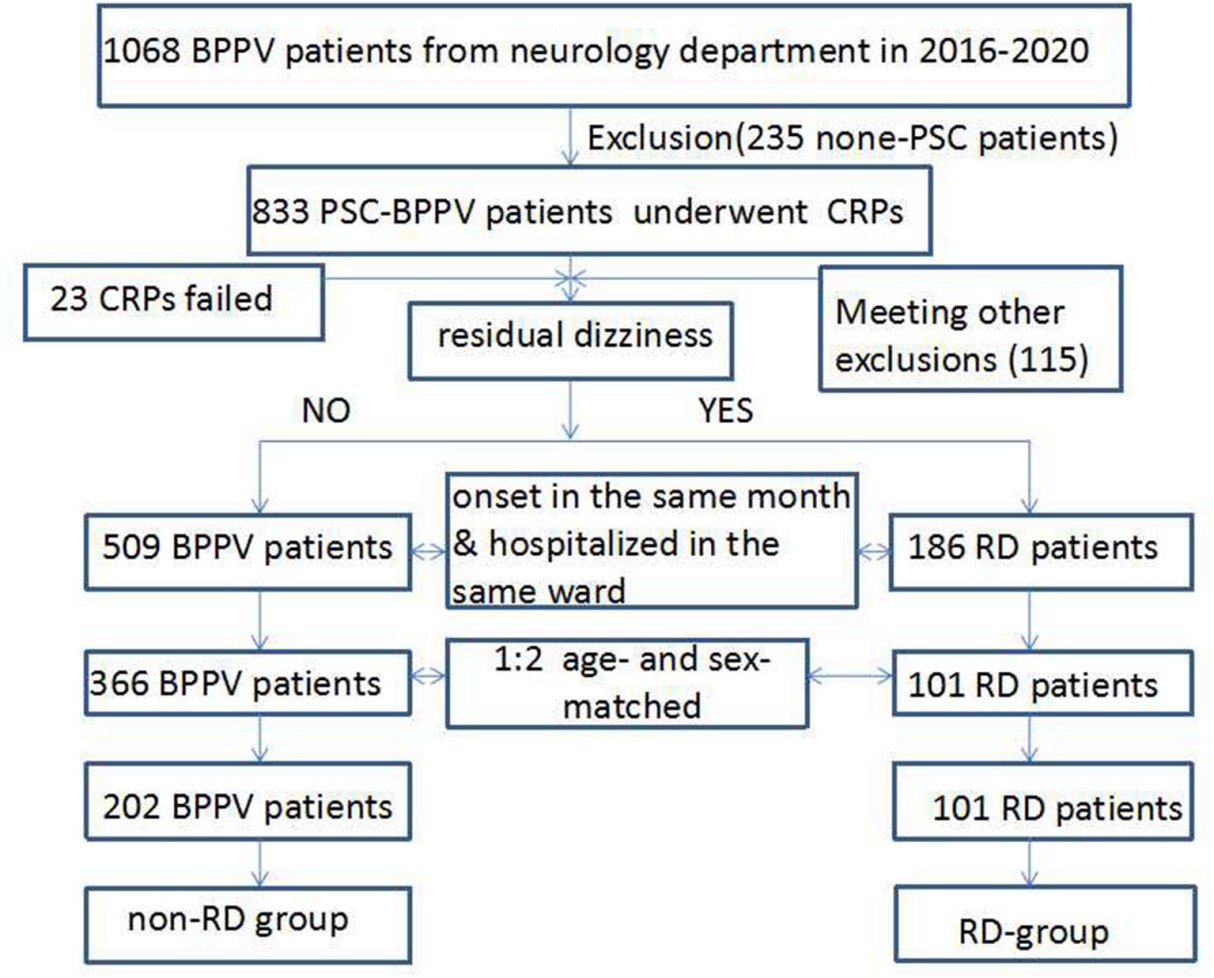
Flow chart for patient enrollment of the retrospective cohort study.

In total, 101 RD subjects (RD group) and 202 age- and sex-matched BPPV patients (non-RD group) were enrolled in the study (Figure 1). We followed the recurrence rate of the enrolled patients at 1, 3, and 6 months. We determined whether there was a recurrence by examining the patient's medical record and confirming the patient's symptoms over the phone.

### Measures

All patients presented to the emergency room with fasting blood collection within 48 h of vertigo. Complete blood counts were determined using the ethylenediaminetetraacetic acid blood sample method on a Toshiba analyzer (Hitachi High Technologies Corporation, Tokyo, Japan). RDW-CV value was calculated as follows: RDW – CV = [red blood cell (RBC) volume standard deviation/average RBC volume] ×100 (13). The normal reference range for RDW in this study was between 11 and 14%. This study reported RDW-CV and denoted RDW.

Meanwhile, serum C-reactive protein (CRP), homocysteine (HCY), albumin (ALB), total bilirubin (TB) levels, alanine aminotransferase (ALT), fasting blood glucose (FBG), uric acid (UA), and creatinine (CRE) were measured by a Hitachi LST008 Analyzer (Hitachi High-Tech, Tokyo, Japan). The values of total cholesterol (TC), triglycerides (TG), high-density lipoprotein cholesterol (HDL), and low-density lipoprotein cholesterol (LDL) were measured with the same analyzer. In addition, fibrinogen (FIB) was measured using an automated coagulation analyzer (ACL-TOP-700, Wolfen, Spain).

### Statistical analysis

Statistical Package for the Social Sciences (SPSS) for Windows (version 24.0. Chicago, IL) was used to describe the enumeration data by the number of cases (constituent ratio); the measurement data was described by the mean ± standard deviation (*x* ± s). Count data were analyzed by chi-square test or Fisher exact test; normally distributed measurement data were analyzed by variance analysis; non-normally distributed data of multiple independent samples were analyzed by Kruskal–Wallis test, and non-normally distributed data of two independent samples were analyzed by Mann–Whitney test. Pearson method was used for correlation analysis of normal distribution materials, and the Spearman method was used for correlation analysis of non-normal distribution materials. Variables with *p* < 0.05 in Table 1 were entered into a forward logistic regression model to identify risk factors for developing RD after BPPV. Results were shown as adjusted ORs (odds ratios) and corresponding 95% confidence intervals (CIs). Receiver operating characteristic (ROC) curve analysis was used to evaluate the ability of RDW to predict RD. The optimal diagnostic cutoff point for RDW was recorded when the Youden index was maximal, and the sensitivity and specificity were calculated separately. Statistical significance was set at *p* < 0.05.

**Table 1:** The clinical characteristics of the study samples.

**Characteristics**	**None-RD (*n* = 202)**	**RD** **(*n* = 101)**	***p-*Value**
Age (years), mean (SD)	58.34 (10.06)	58.34 (10.06)	—
Male, *n* (%)	116 (58)	58 (58)	—
Alcohol consumption, *n* (%)	33 (17)	16 (16)	0.912
Current smoking, *n* (%)	76 (38)	33 (33)	0.236
Hypertension, *n* (%)	64 (32)	27 (27)	0.226
Diabetes mellitus, *n* (%)	42 (21)	38 (38)	0.002^*^
BMI, mean (SD)	24.29 (3.42)	24.27 (3.14)	0.862
SBP (mm Hg), mean (SD)	136.56 (21.99)	134.80 (22.33)	0.360
DBP (mm Hg), mean (SD)	83.60 (12.66)	85.21 (13.52)	0.533
WBC (10^9^/Ul), mean (SD)	7.16 (2.05)	7.50 (1.98)	0.088
N/L, median (IQR)	2.59 (2.43)	2.71 (1.48)	0.006^**^
HGB (10^9^/L), mean (SD)	140.90 (13.49)	142.26 (15.63)	0.464
RBC(10^9^/L), mean (SD)	4.68 (0.56)	4.74 (0.47)	0.073
RDW (%) median (IQR)	12.51 (0.78)	13.63 (1.75)	0.000 ^***^
PLT (10^9^ /L), mean (SD)	233.37 (54.72)	236.17 (61.27)	0.615
ALT (U/L), median (IQR)	20.55 (9.88)	20.51 (9.63)	0.877
TB (μmol/L), median (IQR)	13.74 (5.97)	12.19 (4.76)	0.028^*^
CR (μmol/L), median (IQR)	71.93 (16.60)	67.31 (16.26)	0.016
CRP (mg/L) median (IQR)	4.63 (3.80)	5.96 (3.62)	0.003^**^
FIB (g/L), median (IQR)	2.64 (0.72)	2.79 (0.79)	0.126
ALB (g/L), median (IQR)	42.11 (3.06)	40.85 (3.08)	0.001 ^***^
UA (μmol/L), median (IQR)	375.03 (100.88)	351.69 (92.90)	0.043
TG (mmol/L), mean (SD)	1.54 (0.76)	1.66 (0.87)	0.212
TC (mmol/L), mean (SD)	4.99 (1.06)	5.08 (1.18)	0.741
LDL (mmol/L), mean (SD)	3.06 (0.90)	3.20 (1.04)	0.379
HDL (mmol/L), mean (SD)	1.28 (0.27)	1.25 (0.27)	0.190
FBG (mmol/L), mean (SD)	7.27 (3.00)	8 (1.79)	0.000^***^
Time from onset to hospital (h), median (IQR)	20.04 (22.33)	21.00 (21.15)	0.116
6-month recurrence rate, *n* (%) mean (SD)	32 (16)	38 (38)	0.000^***^
Number of CRPs, median (IQR)	1 (1–5)	2 (1–5)	0.000^***^
**Medication history**			
Antihypertensive therapy, *n* (%)	52 (81)	23 (85)	0.452
Antiglycemic therapy, *n* (%)	26 (62)	15 (40)	0.037^*^

BMI, body mass index, defined as weight in kilograms divided by the square of height in meters; SBP, systolic blood pressure; DBP, diastolic blood pressure; WBC, white blood cells; N/L :Neutrophilic /lymphocytes; HGB, hemoglobin; RBC, red blood cell; RDW, red blood cell distribution width; PLT, blood platelet; ALT, alanine transaminase; TB, total bilirubin; CR, creatinine; CRP, c reactive protein; FIB, fibrinogen; ALB, albumin; UA, uric acid; TG, triglyceride; TC, total cholesterol; LDL, low-density lipoprotein cholesterol; HDL, high-density lipoprotein cholesterol; FBG, fasting blood-glucose.

The differences were considered significant if p-value < 0.05.

*** p-value < 0.001.

** p-value < 0.01.

* p-value < 0.05.

### Ethical considerations

The data and samples analyzed in this study were obtained according to the standards and approval of the Ethics Committee of Zhuhai Hospital of Integrated Traditional Chinese and Western Medicine. Because this study was retrospective and all patient data were analyzed anonymously, the Ethics Committee waived the informed consent of the participants.

## Results

### General statistics

Comparative analysis of clinical characteristics of RD and non-RD patients is shown in Table 1. In terms of personal medical history, there were no differences between the two groups in alcohol consumption, smoking habit, and hypertension (*p* = 0.912, 0.236, and 0.226, respectively). No differences were observed with respect to BMI, blood pressure, RBC, HGB, PLT, ALT, TG, TC, LDL, HDL, or FIB (all *p* > 0.05). RD group had more diabetes and higher fasting blood glucose (FBG) than the non-RD group (*p* = 0.002 and *p* < 0.001, respectively). Regarding the duration of symptoms, the RD group had a longer time from onset to treatment (20.04 ± 22.33 vs. 21.00 ± 21.15, *p* =0.116). There was a significant difference in the recurrence between RD and non-RD groups (*p* < 0.001). Compared with the non-RD group, the number of CRPs was significantly higher (*p* < 0.001) in the RD group.

### OS and inflammatory markers

Higher levels of OS and inflammatory markers in RD patients compared to non-RD patients (*p* < 0.001). Serum levels of UA, TB, ALB, and CRE in patients with RD were lower than those in the non-RD group (all *p* < 0.05), indicating a lower antioxidant status at hospital admission (11). In terms of non-specific inflammatory markers, RD patients had higher levels of N/L and CRP (*p* = 0.006 and 0.003, respectively).

### Spearman's correlation analysis about OS and inflammatory markers

**Figure 2:**
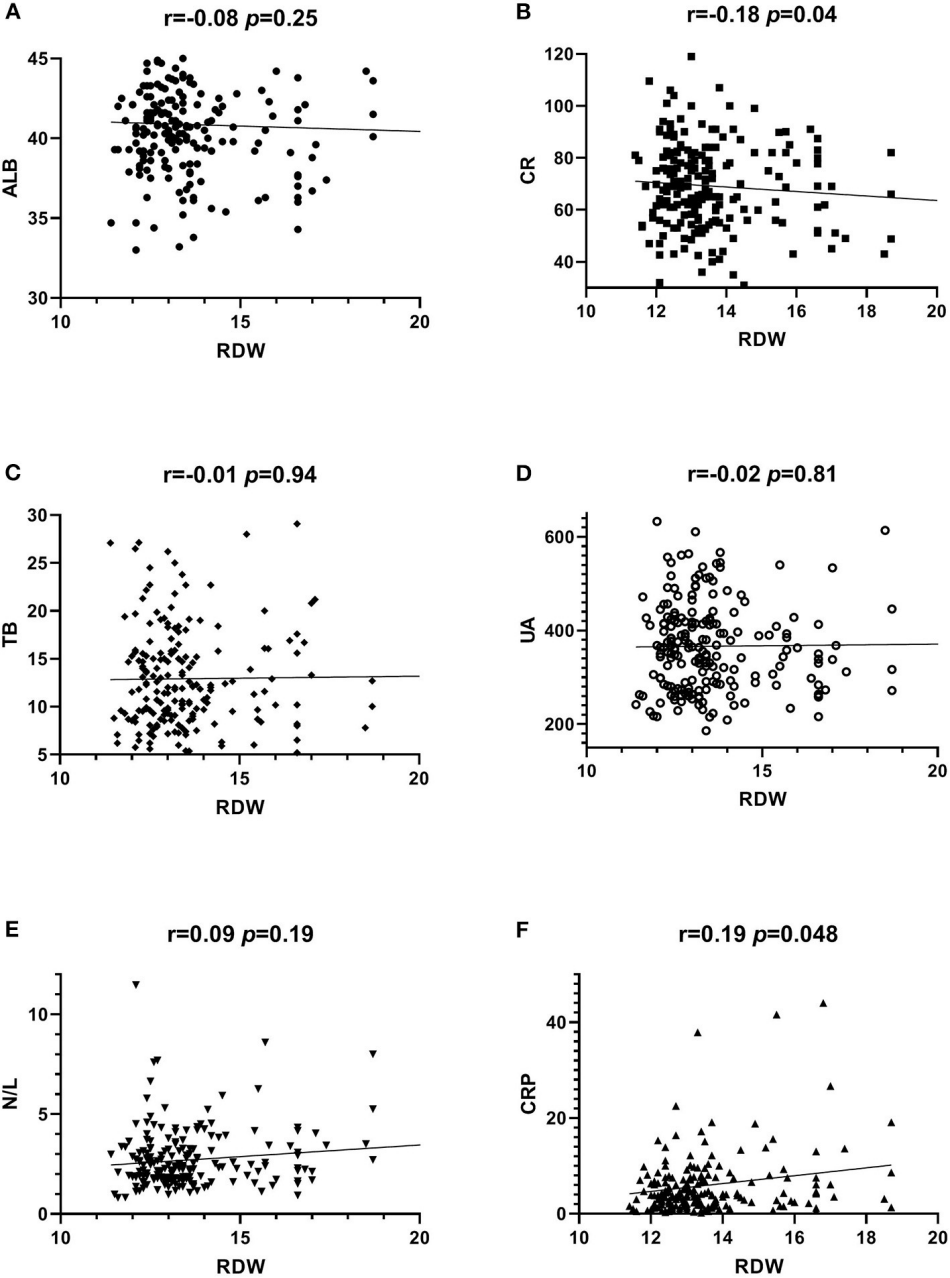
Correlation between serum RDW level and ALB, CR, TB, UA, CRP and N/L in RD patients.

Figure 2 shows the results of Spearman's correlation between OS and inflammatory markers and RD in BPPV patients. RDW was positively correlated with CRP and negatively correlated with CRE (both *p* < 0.05). However, RDW had no correlation with ALB, TB, UA, or N/L (all *p* > 0.05).

### The relationship between RDW value and the number of CRPs

**Figure 3:**
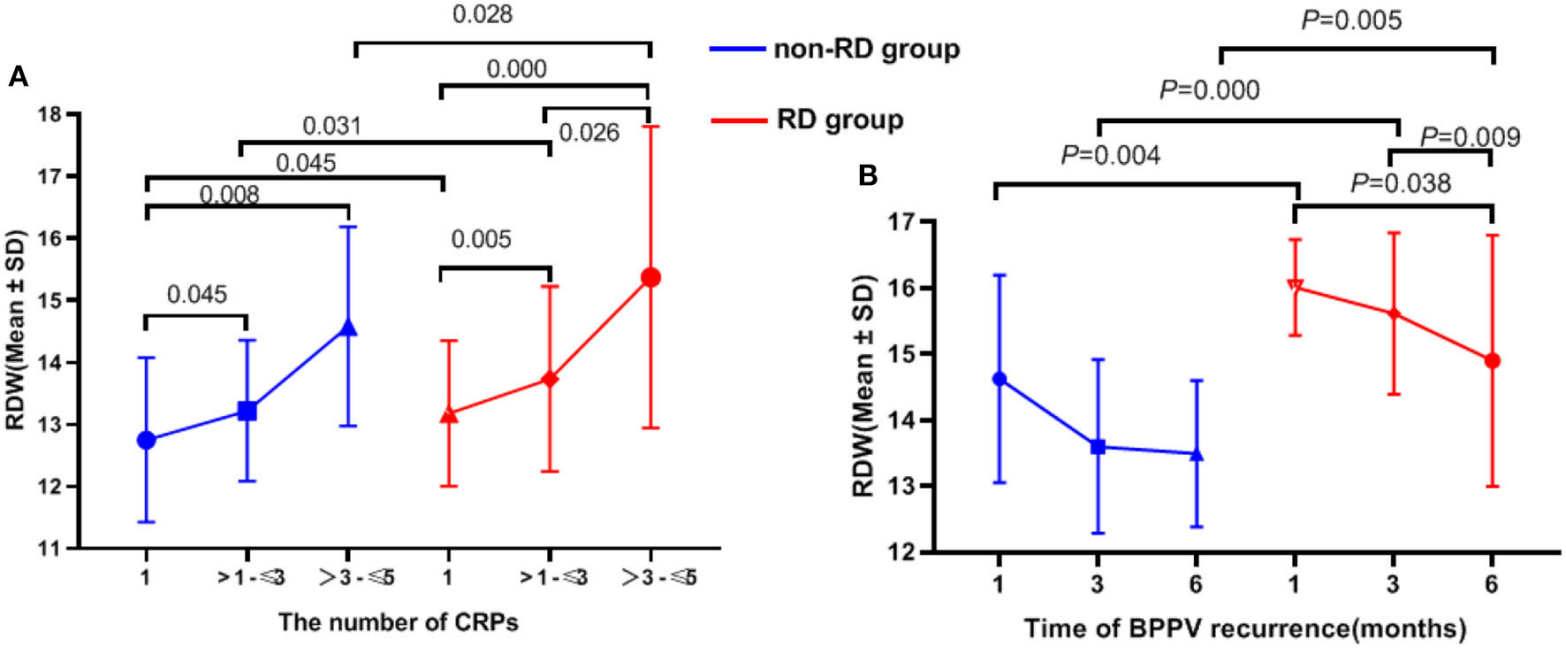
The relationship between RDW value and number of CRPs, recurrence of BPPV in 6 months of follow-up.

The effects of RDW on the number of CRPs between the non-RD and RD group were determined. The results showed significant differences in serum RDW levels between the number of CRPs in both inter-group and intra-group comparisons (all *p* < 0.05). In all, our results showed the number of CRPs in both groups increased with RDW values (Table 2, Figure 3A).

**Table 2:** The relationship between RDW value and number of CRPs, and recurrence of BPPV in 6 months of follow-up.

	**Non-RD group**	**RD group**	***p*-Value**
**The number of CRPs**			
1 median (SD)	12.79 (0.69)	13.18 (1.17)	0.045^*^
>1– ≤ 3 median (SD)	13.25 (0.62)	13.82 (1.94)	0.031^*^
>3– ≤ 5 median (SD)	14.63 (0.77)	15.37 (1.43)	0.028^*^
**Recurrence rate**			
1-month median (SD)	14.74 (1.57)	16.01 (0.73)	0.004^**^
3-month median (SD)	13.60 (1.31)	15.61 (1.22)	0.000^***^
6-month median (SD)	13.49 (1.11)	14.90 (1.90)	0.005^**^

The differences were considered significant if p-value < 0.05.

*** p-value < 0.001.

** p-value < 0.01.

* p-value < 0.05.

### The relationship between RDW value and recurrence of BPPV in 6 months of follow-up

By categorizing the time of recurrence, it can be concluded that the higher the baseline RDW, the earlier the recurrence onset (although not all *p* < 0.05; Table 2, Figure 3B).

### Multivariate analysis

In the multivariate analysis (Table 3), elevated RDW levels was demonstrated as an independent risk factor which may predict prognosis for patients with RD (OR = 2.62; 95% CI, 1.88–3.64; *p* < 0.001).

**Table 3:** Risk factors for RD using multiple logistic regression.

**Risk factors**	**OR**	**95% CI**	***p-*Value**
RDW	2.615	1.875–3.648	0.000^***^
CR	1.048	1.018–1.079	0.002^**^
ALB	0.847	0.770–0.931	0.001^**^
TB	1.082	1.022–1.145	0.007^**^
GLU	1.327	1.143–1.542	0.000^***^

The differences were considered significant if p-value < 0.05.

*** p-value < 0.001.

** p-value < 0.01.

* p-value < 0.05.

### ROC analyses

**Figure 4:**
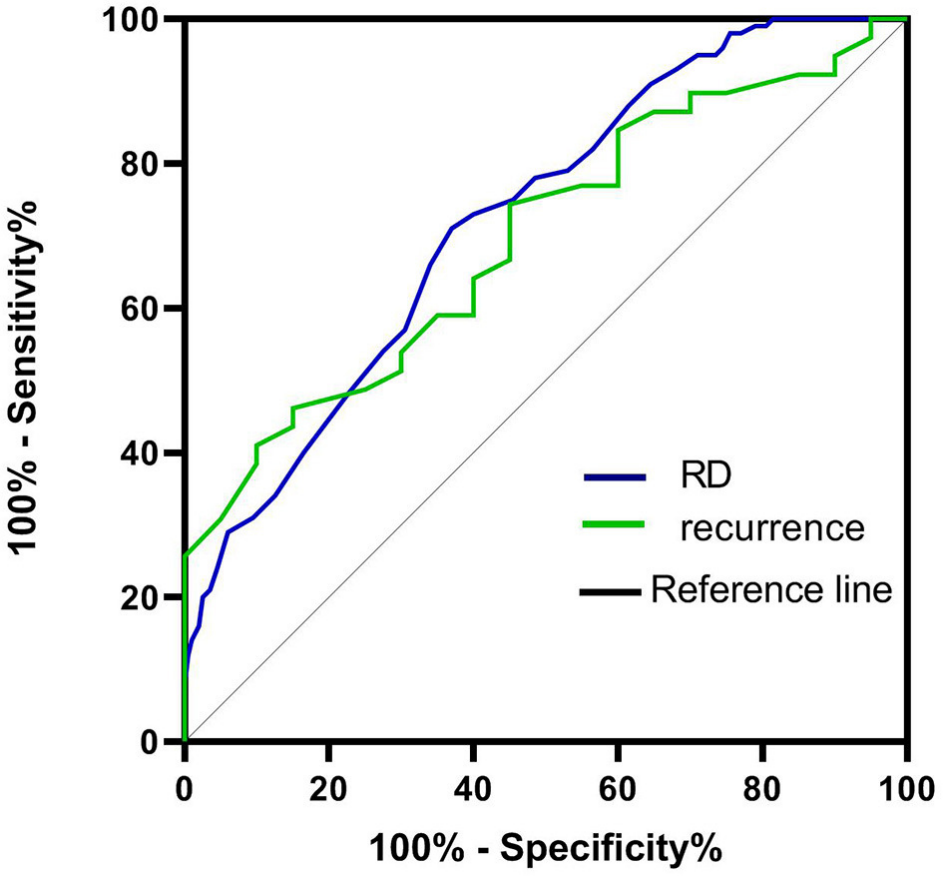
ROC curve to establish the sensitivity and specificity of RDW levels to predict the risk of RD and recurrence after BPPV.

According to the ROC curve analysis, the optimal cutoff value of the RDW that best distinguished the presence of RD was 12.95%. The area under the curve (AUCs) for the ability of the RDW to predict RD was 0.723 with 75.6% sensitivity and 69.5% specificity, respectively. The AUC for the ability of the RDW to predict recurrence was 0.692 (95% CI = 0.561–0.831; *p* < 0.014), and the optimal cutoff value was 13.45% (Figure 4)

## Discussion

Our results suggested that (1) RDW was significantly higher in the RD group than in non-RD group, (2) RDW was independently associated with RD-causing 2.62-fold risk increase for every one unit increase in RDW value, (3) a cutoff value of 12.95 for RDW with a sensitivity of 75.6% and a specificity of 69.5% was obtained in the ROC analyses. To our knowledge, this was the first study to investigate the association of RD with RDW, and the first report on RDW as indicators to evaluate the OS status of RD.

Otolithic organ disorder (OOD) is considered the most studied and persuasive among the hypotheses of RD's occurrence and recurrence (21). Emerging literature has shown that patients with BPPV had higher levels of OS and subsequent elevated inflammatory responses, which may contribute to the development of OOD (22, 23). Other studies concluded that OS caused damage to vestibular hair cells and neurons in the inner ear, impairing vestibular function (19). Sequentially, we hypothesized that patients with BPPV were more prone to RD after recovery of CRPs if accompanied by higher OS.

### RDW and OS

Cumulative evidence indicated that serum levels of oxidants increased with RDW (18, 24). In this study, the RDW in RD patients was significantly higher than that in non-RD subjects, indicating that RD incidence in BPPV patients may be caused by higher OS. The antioxidant and oxidant systems were imbalanced. Theoretically, low serum antioxidant concentrations may be inversely associated with RDW. Some scholars have found that UA, TB, ALB, and CR can comprehensively reflect the antioxidant status of patients with BPPV (11). Our study found that these four indicators were lower in the RD group than in non-RD group, suggesting that RD patients have lower antioxidant capacity. Moreover, RDW values were inversely correlated with the levels of these four markers (although not all findings were statistically significant), which also indicated that RD patients had weaker antioxidant capacity.

The imbalance between antioxidant and oxidant causes oxidative damage, which can lead to OOD. OS occurs under conditions of increased reactive oxygen species or depletion of antioxidants. Scholars investigated the relationship between antioxidants (serum selenium) and RDW and found that patients with higher RDW had lower serum selenium levels (25). Activation of OS and reduction or depletion of endogenous antioxidant activity promote the increase of RDW.

As we all know, OS plays an important role in several inner ear diseases and normal aging (26). Many studies have shown that the incidence of RD was higher with increasing age, which also proves from another perspective that the etiology of RD may be the result of long-term OS accumulation (27, 28). It is well known that RDW levels increase with age (29), which proves that RDW is an indicator of OS in terms of physiological degradation. In our study, higher OS was a risk factor for the development of RD, suggesting an important role of OS in the pathogenesis. The reasons behind this interesting result deserve further investigation.

### RDW and OS-related inflammation

Researchers have found that serum levels of inflammation increased with RDW (25). OS can decrease the lifespan of erythrocytes, while subsequent inflammation is strongly associated with inhibited erythropoiesis, both of which may increase RDW level (24). Scholars investigated the relationship between inflammation markers and RDW and found that patients with higher RDW had higher levels of N/L and IL-6 (25, 30). Similarly, in our study, we found higher levels of RDW, N/L, and CRP in the RD group, which were statistically different from the non-RD group. We excluded infectious diseases and compared WBCs between the two groups to make sure the data were accurate. Given this premise, RDW was positively correlated with CRP in this study, suggesting that RD patients suffered a more severe inflammatory response.

The inflammation cascade reactions may be involved in the pathogenesis of the otolith organ (31). The inner ear has a blood-labyrinth barrier, is connected to the cervical lymph nodes, and produces cytokines through the spiral ligament to participate in the inflammatory response (32). Based on the above mechanisms, the link between inflammation and RD will become more apparent as the disease progresses.

Taken together, our study showed that patients with RD had upregulated inflammation, and the OS-related inflammation was a possible mechanism in the pathogenesis of RD.

### RDW and microcirculation

Oxidative stress is a critical factor that causes microcirculation disturbance (33). OS induces erythrocyte adhesion to the vascular endothelium and reduces erythrocyte deformability (34). Moreover, the elevated RDW promotes platelet activation and aggregation, which serves as a marker of the procoagulant status of RBCs (35). The two interact with each other and play a role in the elevation of vessel resistance, which would deform worse and impair blood flow through microcirculation. Increased baseline RDW has been shown to be associated with poor collateral flow in large artery atherosclerosis stroke patients (36). The labyrinthine artery, as the only artery supplying the vestibular system, has less collateral circulation. Therefore, it is reasonable to believe that when RDW increases, its microcirculation becomes poorer and OS is more severe.

The present study revealed that FIB levels were elevated in RD group, indicating higher procoagulant status and worse microcirculation. This is in agreement with a previous study focused on microcirculation, which found that elevated RDW led to slow coronary flow (37). Apart from continuous degradation of the vestibule caused by OS, the deterioration of microcirculation caused by OS may be another reason for the high incidence of RD in older adults. Deng et al. found that Danhong injection significantly improved RD through antioxidant activity and microcirculation improvement (38). However, their results were speculative theories that were supported mostly by pathophysiologic reasoning.

### RDW value and the recurrence rate of BPPV and number of CRPs

OS is related to OOD, and it could also be related to its recurrence rate and not only with RD. In this study, the recurrence rate of BPPV was higher in the RD group as compared to the non-RD group, suggesting an association between RDW and recurrence (Table 1). As shown in Figure 3, it can be concluded that the higher the baseline RDW, the earlier the recurrence onset (although not all *p* < 0.05; Table 2, Figure 3B). Moreover, the AUC for the ability of the RDW to predict recurrence was 0.692. In terms of the number of CRPs, our results showed that RDW values in the two groups increased with CRPs (all *p* < 0.05; Table 2, Figure 3A). The number of CRPs was proportional to the RDW value. The reasons behind the interesting results deserve further investigation.

As mentioned above, the results of this study showed a higher RDW in the RD group, indicating a higher level of OS in RD patients. In addition, we corroborated the lower antioxidant levels of RD patients by their serum levels of UA, TB, ALB, and CR. In conclusion, our study shows that BPPV patients with a higher OS are more prone to develop RD, which may help to evaluate the prognosis of BPPV patients.

### Limitation

Our study has the following limitations. First, the one-time measurement of the RDW value was prone to lead to analytical errors. Second, this was a retrospective study, and the sample was relatively small, which may bias the findings. Third, several OS markers (such as MDA and SOD) and vestibular function test (VEMP and SVV) were not adequately evaluated.

## Conclusions

To sum up, our result suggested that RDW could be considered as potential biomarkers of RD. As it is a rapid, inexpensive, and easily available laboratory marker, it can be used in clinical practice for prediction of RD. Also, we put forward a hypothesis that OS plays a role in RD, which needs further investigations.

## Data availability statement

The raw data supporting the conclusions of this article will be made available by the authors, without undue reservation.

## Ethics statement

The studies involving human participants were reviewed and approved by Zhuhai Hospital of Integrated Traditional Chinese and Western Medicine. Written informed consent for participation was not required for this study in accordance with the national legislation and the institutional requirements.

## Author contributions

Conceptualization, data curation, and writing review and editing: K-HX. Formal analysis: L-LL. Investigation and writing original draft: L-CC. Project administration: C-YS and HL. Resources: Q-QC. Software: R-NL. Supervision: W-KH. Validation: L-CC. Visualization: J-SH. All authors contributed to the article and approved the submitted version.

## Conflict of interest

The authors declare that the research was conducted in the absence of any commercial or financial relationships that could be construed as a potential conflict of interest.

## Publisher's note

All claims expressed in this article are solely those of the authors and do not necessarily represent those of their affiliated organizations, or those of the publisher, the editors and the reviewers. Any product that may be evaluated in this article, or claim that may be made by its manufacturer, is not guaranteed or endorsed by the publisher.
